# Biomaterials for extrusion-based bioprinting and biomedical applications

**DOI:** 10.3389/fbioe.2024.1393641

**Published:** 2024-06-21

**Authors:** Arianna Rossi, Teresa Pescara, Alberto Maria Gambelli, Francesco Gaggia, Amish Asthana, Quentin Perrier, Giuseppe Basta, Michele Moretti, Nicola Senin, Federico Rossi, Giuseppe Orlando, Riccardo Calafiore

**Affiliations:** ^1^ Smart Manufacturing Laboratory, Engineering Department, University of Perugia, Perugia, Italy; ^2^ Laboratory for Endocrine Cell Transplant and Biohybrid Organs, Department of Medicine and Surgery, University of Perugia, Perugia, Italy; ^3^ Department of Civil and Environmental Engineering, University of Perugia, Perugia, Italy; ^4^ Wake Forest School of Medicine, Winston Salem, NC, United States; ^5^ Engineering Department, University of Perugia, Perugia, Italy; ^6^ Diabetes Research Foundation, Confindustria Umbria, Perugia, Italy

**Keywords:** bioprinting, material extrusion, biomaterials, biomedical applications, artificial tissues

## Abstract

Amongst the range of bioprinting technologies currently available, bioprinting by material extrusion is gaining increasing popularity due to accessibility, low cost, and the absence of energy sources, such as lasers, which may significantly damage the cells. New applications of extrusion-based bioprinting are systematically emerging in the biomedical field in relation to tissue and organ fabrication. Extrusion-based bioprinting presents a series of specific challenges in relation to achievable resolutions, accuracy and speed. Resolution and accuracy in particular are of paramount importance for the realization of microstructures (for example, vascularization) within tissues and organs. Another major theme of research is cell survival and functional preservation, as extruded bioinks have cells subjected to considerable shear stresses as they travel through the extrusion apparatus. Here, an overview of the main available extrusion-based printing technologies and related families of bioprinting materials (bioinks) is provided. The main challenges related to achieving resolution and accuracy whilst assuring cell viability and function are discussed in relation to specific application contexts in the field of tissue and organ fabrication.

## 1 Introduction

The term “bioprinting” indicates the use of additive manufacturing (AM) technologies to produce bio-engineered structures for medical and biotechnology applications, often mimicking biological tissues, organs and cells (Moroni et al., 2018; Rider et al., 2018). In AM, parts are fabricated by gradually adding material. The vast majority of AM technologies adopt a “layer-based” manufacturing approach, where a part is built by depositing (i.e., “printing”) material into a series of thin, 2D horizontal patterns laying on top of each other (International Organization for Standardization, 2021). Through the deposition of multiple layers, entire 3D parts can be created (hence AM is sometimes also referred to as “3D printing”). In bioprinting, bespoke AM technologies are used, designed to print (again, most often in a layerwise fashion) biomaterials that often contain living cells, or biomaterials designed to accommodate cells added post-print. The term “biomaterial” *per se* refers to any synthetic substance engineered to interact with a living system (or to a natural substance compatible with a living system) (Williams, 2009; Groll et al., 2016). When biomaterials are merged with living cells before they are printed, they are referred to as “cell-laden biomaterials” and the main challenge is to ensure the cells survive the printing process. If cells are to be added post-print, then the main challenge is to ensure that cells are able to populate the construct and flourish within it.

The main additive manufacturing technologies adopted in bioprinting are related to the different ways a biomaterial (cell-laden or not) can be deposited and held in place onto a layer. The main technologies are (Rider et al., 2018; International Organization for Standardization, 2021).

- material-jetting (also known as: inkjet-based), where droplets are deposited using mechanisms similar to those implemented in desktop inkjet printers. The material is forced through orifices using pressure pulses produced by piezoelectric, thermal or solenoid controllers (Guo et al., 2017);
- laser-assisted, where material droplets are moved using a laser source from a donor material to a receiver material (a biopolymer or a cell culture medium) in predetermined locations (Guillotin et al., 2010);
- vat-photopolymerization, or stereolithography, where a photosensitive polymer contained in a vat is selectively solidified upon illumination (International Organization for Standardization, 2021); and
- extrusion based, where the material is extruded through a nozzle, orifice or needle, using different systems, i.e., pneumatic, screw-based or piston-based (see section 4.1.1) (Rider et al., 2018).

All the above families of technologies are characterized by different challenges involving the possibility to accommodate cell-laden materials as opposed to adding cells post-print, cell survivability, capability of realizing cellular and extra-cellular microstructures at the required accuracies and resolutions, capability of ultimately realizing tissues or organs that may act as consistent reference models for medical/pharmaceutical experimentation, or may act as valid supports for regenerative medicine, or may even act as entire replacements of natural tissues or organs.

Amongst the available bioprinting technologies, this paper focuses on material extrusion (International Organization for Standardization, 2021). Extrusion-based bioprinting (often referred to as MEX-bioprinting in short form) is most commonly implemented as described above, i.e., by pressing a material through a nozzle (orifice or needle) by mechanical means and having it deposit onto a solid substrate. However, further variations of MEX-bioprinting include also.

• Coaxial bioprinting: achieved through the use of two or more independent nozzles arranged in a coaxial, concentric configuration (in the most typical architecture a central nozzle extrudes one material acting as core, and a second nozzle, with annular opening around the central nozzle, extrudes a second material that forms an outer shell around the core). Coaxial bioprinting is used to create complex constructs including cell-laden bioinks, crosslinkers and sacrificial material. The multi-material combination allows for the fabrication of hollow tubular structures, e.g., vascular networks, and solid fibers (Mohan et al., 2022);
• FRESH bioprinting: Freeform Reversible Embedding of Suspended Hydrogels bioprinting. Consists of using a syringe-based extrusion system to deposit material into a biocompatible sacrificial support bath. By depositing into a bath as opposed to on a solid surface, FRESH bioprinting counterbalances gravity effects which would hinder the deposition of specific types of low-viscosity bioinks (Hinton et al., 2015; Shiwarski et al., 2021);
• Microfluidic bioprinting: implements extrusion by using microfluidic devices. By extruding bioinks through microchannels, it is possible to better control the deposition, leading to higher geometrical accuracy. The use of microfluidic devices also allows for the simultaneous use of different materials and eases switching bioinks during the fabrication, which makes possible the creation of complex patterns and the differentiation of mechanical properties within the same print job (Costantini et al., 2017; Chatinier et al., 2021).

In general, thanks to the possibility of using diverse materials and technologies, the family of MEX-based bioprinting processes holds great potential for different applications, in particular for developing tissues for drug development, manufacturing human organs, easing tissue regeneration and studying cellular mechanisms (Murphy and Atala, 2014; Seol et al., 2014; Yi et al., 2021). This paper focuses on the fabrication of tissues and organs and illustrates examples involving the fabrication of diverse tissues and cell types; namely, bone, cartilage, liver, nervous and skin.

### 1.1 Historical background

**FIGURE 1:**
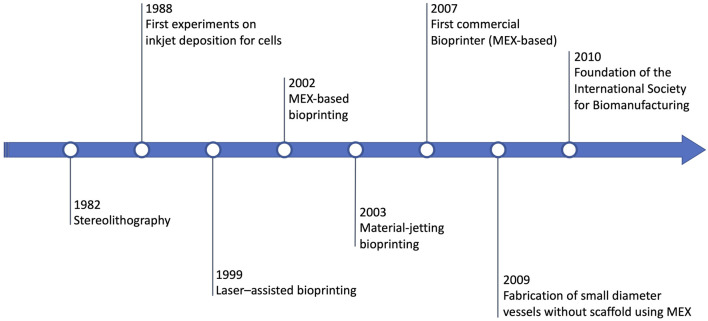
Historical timeline of bioprinting including the milestones of MEX-based bioprinting.

The history of MEX-based bioprinting is briefly summarized in Figure 1. Extrusion-based bioprinting proceeds in parallel with the history of the other bioprinting technologies, and more in general, with the history of additive manufacturing/3D printing as a whole (Xing et al., 2023). The first significant general-purpose 3D printing technology, stereolithography, is introduced by Charles Hull in 1984 (Gu et al., 2020). A few years later, in 1988, the first example of bioprinting is provided by Klebe, who instead uses material-jetting (via a Hewlett-Packard (HP) inkjet printer) to deposit cells (Klebe, 1988). In 1999, yet another technology, i.e., laser–assisted bioprinting, is demonstrated for the production of the first anatomical structures (Odde and Renn, 1999). In 2002, MEX-based bioprinting is demonstrated, along with the introduction of the term “3D Bioplotter” (Landers et al., 2002). One year later, the first bioprinter based on dedicated material-jetting technology is developed (Wilson and Boland, 2003; Roth et al., 2004). Soon after, the first bioprinting company (Organovo, founded in 2007) launches the first commercial bioprinter in 2009, based on material extrusion (Thayer, Martinez, and Gatenholm, 2020). In 2009, vascular cells (as agarose) are used to re-generate small diameter vessels without scaffolds, again using material extrusion (Norotte et al., 2009). One year later, the International Society for Biomanufacturing is founded, while in 2011 the Biomedical Research Institute of Hasselt University (Belgium) manufactures a metal mandible (Mei et al., 2021), a fundamental step towards bioprinting achieving official recognition in clinical settings.

Several medical and biotechnology application fields have been benefiting from the introduction of bioprinting. As stated earlier, this paper covers the fabrication of tissues and organs. The regenerative capacity of tissues and, more in general, organs, is limited for humans (Tang et al., 2019): in case of organ failure, the last treatment possible is transplantation, but the waiting list is drastically long and only a low percentage of patients can effectively benefit from it (Xing et al., 2023), moreover, the risk of incompatibility is concrete. All such issues justify the investments to compensate for the organ source shortage (Abouna, 2008). One of the most promising answers in this sense, is the combination of tissue engineering and regenerative medicine (Berthiaume, Maguire, and Yarmush, 2011). The most widespread approach is based on the usage of scaffolds to repair tissues and organs (Yadid, Feiner, and Dvir, 2019): bioprinting technologies can be used to fabricate scaffolds, as alternative to freeze-drying, electrospinning and others (Fleischer, Feiner, and Dvir, 2017). Scaffolds play the role of supports and also constitute a favourable microenvironment for the growth and survival of cells (You et al., 2011).

Bioprinting of tissues/organs has also been advantageously applied to create models, useful for combating infectious diseases (Chen et al., 2023). In this latter case, bioprinted constructs are used to simulate *in vitro* human tissues and organs attacked by viruses and other pathogens, therefore giving the opportunity of testing therapeutics and vaccines before directly test them in humans (Zimmerling and Chen, 2020; Zimmerling, Zhou, and Chen, 2021). To be suitable for application, bioprinted constructs for tissues and organs must be able to feature required microstructural, mechanical and biological properties (Chen et al., 2023). The external structure must be extremely similar to those of human organs, while the internal must be highly porous, to favour the transport of nutrients and the growth of cells, together with the easy of removal of metabolic wastes. In terms of mechanical properties, these constructs must ensure mechanical strength and biodegradation. At the initial phase, the artificial tissues must provide the required mechanical strength; then, with the biological reformation of tissues, they must biodegrade. The initial mechanical strength of bioprinted tissues must be as much as possible similar to that of the original organic tissue. Moreover, the velocity of degradation should be equal to the growth rate of replacing tissues. Finally, these constructs must ensure the attachment, proliferation and differentiation of cells and, reduce as much as possible negative effects as inflammation and others.

From a historical perspective, early 3D printing approaches dedicated to reproducing tissues or organs could not even produce viable scaffolds, as the initial materials would not be biocompatible. With the advent of biocompatible materials (biomaterials) and the achievement of biodegradability, the first tissue engineering scaffolds were produced, followed by *in vitro* biological models featuring living cells (Rahimnejad et al., 2022), useful to artificially produce different tissues for organs, such as liver and hearth (Zhang et al., 2018). Currently, the target is to directly create living biological structures and micro-organs (Csizmar, Petersburg, and Wagner, 2018), although many challenges remain unsolved, in particular in relation to the intrinsic lack of speed of layer-based manufacturing, and to the still too limited accuracy and resolution in reproducing complicated microstructures with current biomaterials and bioprinting technologies. As expectable, the large-scale production of bioprinted tissues that includes and optimise all these properties, is extremely complex and requires further investments and scientific efforts to be widely accessible and completely mature.

### 1.2 Structure of this review

In the first section, biomaterials are discussed. The paper proceeds with the description of MEX-based bioprinting technologies and the most relevant printer models. Finally, the main applications related to tissue and organ bioprinting are illustrated and discussed.

## 2 Biomaterials for biomedical applications

In this section, the main materials used in extrusion-based bioprinting for biomedical applications are discussed. It should be noted that many of the materials illustrated in the following are also often found in combination to other bioprinting technologies such as, for example, material-jetting. The frequent overlap between materials and technologies is also hinted at by the word “bioink” being frequently used to describe any biomaterial suitable for being printed with any bioprinting technology, although strictly speaking the term refers to inkjetting, and thus should be reserved to refer to biomaterials whose rheological properties make them best suited for material-jetting technologies (Guo et al., 2017).

**FIGURE 2:**
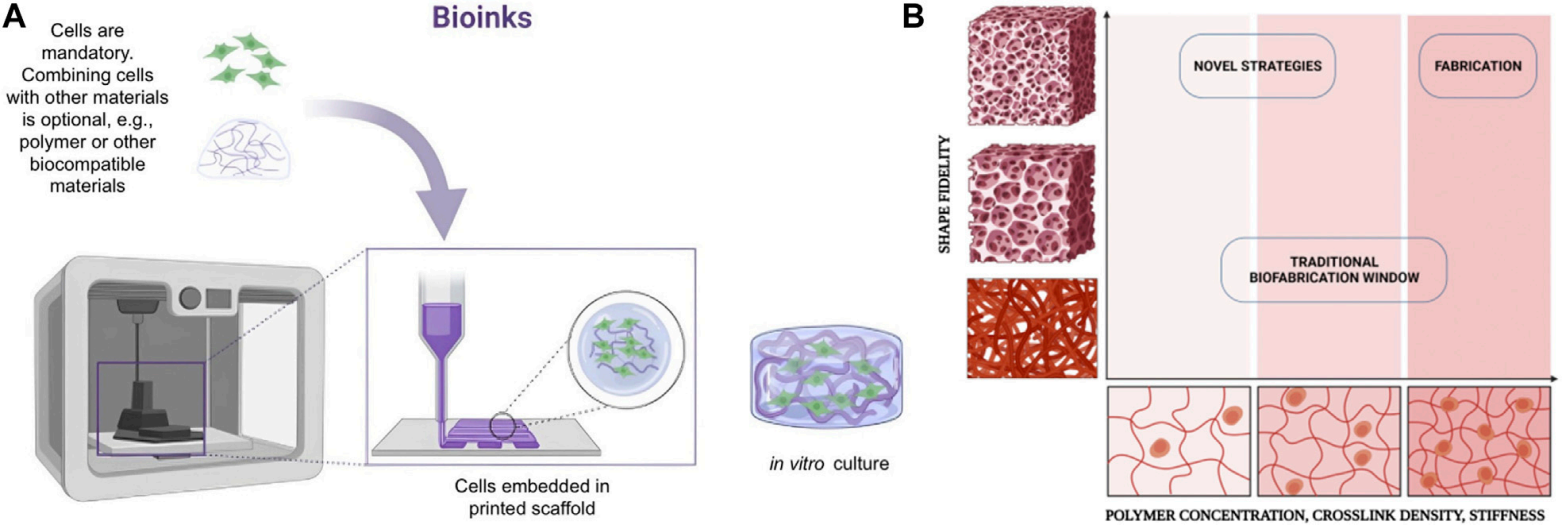
**(A)** Biocompatible materials should be easily extruded and cast into well plates and other moulds. **(B)** Schematic representation of the biofabrication window with the relation between shape fidelity and polymer concentration, cross-linking density and stiffness. Created with BioRender.com.

Generally, biomaterials/bioinks for bioprinting are based on a “matrix” or “support” material, usually a natural or synthetic polymer, to which living cells are added before-print (i.e., cell-laden bioinks), although the addition of living cells post-print is also a possibility. Many of the polymeric materials useable to make bioinks are classifiable as “hydrogels”. Hydrogels are mixtures of a solid phase (porous and permeable) and a liquid one (often water). In cell-laden biomaterials, the hydrogel “captures” the cells and acts as a support and transport medium during and after the printing process. For use with MEX-based bioprinting, hydrogels are typically designed to achieve rheological properties that favour the extrusion process, i.e., they must be capable of flowing with ease through the extrusion nozzle, orifice or needle and must be castable on flat surfaces or other moulds. After the printing process, printed hydrogels must be capable to remain in the shape created by printing with the required accuracy, resolution and stability. The hydrogel must also contribute to ensuring that the cells survive the printing phase and remain viable enough to allow for post-print culturing (see Figure 2). The development of bioinks for extrusion-based bioprinting is therefore characterised by the simultaneous need to address different challenges related to accuracy, resolution and stability of the printed constructs, printability (in this case, specifically in relation to material extrusion technologies) and cell viability (Chimene et al., 2016; Jang et al., 2018).

The terms “structural” or “functional” are often used in combination with bioinks to highlight specific properties. “Structural” bioinks feature biomaterials specifically designed to achieve robust and stable geometric structures. Examples are materials like alginate, decellularised ECM and gelatines. Through the choice of biomaterial, one can control mechanical properties, shape and size, whilst keeping track of cell viability (Song et al., 2011; Gungor-Ozkerim et al., 2018). The term “functional bioink” is instead usually reserved to describe biomaterials have been designed to promote and control cellular growth, development, and differentiation within the bioink. Cellular growth can be achieved by integrating growth factors, and/or by implementing specific surface texture and shape in the structural biomaterial featured in the bioink. The geometry of the printed structure is indeed extremely important in functional bioinks, as it may act as physical cue to promote growth, therefore its design is one of the main challenges in the development of next-generation functional bioinks (Xu et al., 2009; Matai et al., 2020; Sabzevari et al., 2023). The geometry of the printed structure is important also for another two reasons. Bioprinted structures can be extremely fragile and flimsy due to intricate structures and overhangs during printing, therefore additional geometric elements can be created in the structure, with the sole purpose of acting as reinforcers/stabilisers until the printed structure is sufficiently stable and robust to support itself. The second reason of importance of geometry is that additional geometric elements can be embedded in the print, whose purpose is to act as interfaces to other systems used to develop the tissue at a faster rate, or as fixture for inserting the printed construct into a bioreactor (Hollister, 2005; Henmi et al., 2007; Montanucci et al., 2017).

In the following, the main materials typically used to make the matrix/support of printable bioinks for material extrusion are discussed. As commented earlier, many of these materials can be utilised also in combination with inkjet-based or other bioprinting technologies. The following materials are designed to be printed either with or without cells. In the former case, they are the “medium” within which cells must be embedded. Therefore, these materials are supposed to defend the cells (so that they can survive the printing process), transport the cells (through the printing apparatus and to their final localisation in the printed part), and must ultimately allow the cells to “function” post-printing (e.g., proliferate, initiate other biological processes, or simply keep on surviving over a target life span).

### 2.1 Alginate

Alginate is a polyanionic, hydrophilic polysaccharide consisting of linear (1–4)-linked β-D-mannuronic acid (M blocks) and its C5-epimer α-L-guluronic acid (G blocks) residues. The G block content in alginate varies between 30% and 70%. The variability in G block content is mainly function of the species and the seaweed selected for alginate extraction. The G blocks improve the gel production and are separated from M blocks by MG regions. MG and M blocks favour the flexibility of the gel. Due to affinity to alkaline earth metals (with the only exception of Mg^2+^), the presence of divalent cations is responsible for the gelation of alginate via G blocks. The affinity for the cations varies in an increasing manner as Ca^2+^ < Sr^2+^ < Ba^2+^ (Smidsrød, 1970; Smidsrød, 1970; Draget, Smidsrød, and Skjåk-Bræk, 2005; Mørch et al., 2006; Montanucci et al., 2015). Further research efforts are required to better undersand the alginate gelation mechanisms in presence of divalent cations. However, it is now proved that gelation occurs in an “egg-box” form, where ions are trapped between a couple of surrounding helical chains. Calcium alginate-based hydrogel is the most widespread and explored hydrogel system for 3D bioprinting, as it features high performance both in terms of printability and versatility. Alginate has been widely used in biomedicine because of its biocompatibility, low cytotoxicity, mild gelation process, formability and low cost even if it had low viscosity (Gao et al., 2017). The United States Food and Drug Administration (FDA) has also approved the use of some alginates in food. Concerning bioprinting, alginates are considered particularly suitable due to their mild cross-linking conditions via incorporation of divalent ions such as calcium.

The bioprinting technologies most frequently used with alginates are extrusion-based and inkjet-based-jetting bioprinting. In order to use alginates to make a bioink, their viscosity must be usually increased (Jia et al., 2014). Alginates performance/gel cross-linking is dependent on molecular weight, loading of the divalent metal salt and its solubility. The properties of alginate gels are often improved by combination with other polysaccharides. Other additives can be added such as nanomaterials to enhance rheological properties, growth factors to promote stem cell differentiation, and peptides to improve cell adhesion. The possibility of adding multiple components into alginate gels gives them considerable potential for further development (Piras and Smith, 2020). Additionally, alginate-based bioinks can be made by blending-in other materials such as nanocellulose, in particular for application to tissues such as cartilage (Markstedt et al., 2015). Blending is usually performed to increase gelation speed of the bioink, which improves printability and quality of the result. Alginate is now considered the most viable natural polymer for 3D bioprinting and is consequently the most selected material for *in vivo* research.

### 2.2 Gellan Gum

Gellan Gum (GG) consists of an anionic polysaccharide having high molecular weight and hydrophilicity. More specifically, GG is a tetrasaccharide repeating unit of α-l-rhamnose (Rhap) (one unit), one β-d-glucuronic acid (GlcpA) (one unit) and two β-d-glucoses (Glcp) (two units) (Jansson, Lindberg, and Sandford, 1983). GG is produced from *Pseudomonas* Elodea. In the food sector, Gellan Gum is widely used as thickening and gelling element. Moreover, it can be also used as stabilizer. Gellan Gum shows high biocompatibily and low cytotoxicity (Oliveira et al., 2010; Silva-Correia et al., 2011). For such reasons, GG is widely used in biomedical studies and applications. Hydrogels based on Gellan Gum are obtained via physical crosslinking, carried out by temperature variation and due to the presence of positive ions. With the decrease of temperature, GG shows a conformational transition between the disordered single-chain state to a more ordered double-helix state. The gelification process was found to depend on the re-organization of the double-helix into three-dimensional structures. Metallic cations are crucial for the aggregations of helices: these compounds are capable to electrically screen the carboxyl groups (Milas, Shi, and Rinaudo, 1990). Like Alginates, also GG is approved for use in food by the United States FDA. In relation to printability, GG behaves similarly to alginate. Notably for bioprinting applications, GG can form a hydrogel at low temperatures, although it is rarely used alone for bioprinting purposes. Conversely, GG is mainly used as a gelling agent and stabilizer specifically when added to Poly (d,l-lactide-co-glycolide) (PLGA), also used for blending different bioinks, proved, for instance, useful for the culture of chondrocytes (Pitarresi et al., 2020). The constantly growing interest in enhancing GG bioactivity, has led to the production of bioinks that combine GG with poly-ε-lysine. This latter compound consists of a natural homo-poly-amino acid, which favors the non-specific cell adhesion (Duffy et al., 2021). Inkjet printing has been employed to fabricate cell-embedded patterns like 3D corneal constructs. It has been reported that GG solutions of concentrations above 0.5% are printable only above room temperature. Poly-ε-lysine/GG bioinks have yielded translucent hydrogel 3D structures with micron-scale resolution, biocompatible with corneal epithelial and endothelial cells.

### 2.3 Agarose

Agarose (AG) is a natural linear polysaccharide extracted from marine algae and red seaweed. AG features d-galactose and 3,6-anhydro-l-galactopyranose, repeated polymer chains connected by glycosidic bonds (α-1,3 and β-1,4). AG is commonly used in electrophoresis applications as well as tissue engineering for its gelling properties. The thermosensitive properties of low-melting temperature AG enable it to rapidly transition into a gel state after printing, which is a property that makes it attractive for use in bioinks (Ozbolat and Hospodiuk, 2016). The melting and gelling temperatures of agarose can be modified chemically, which widens the printability window, as a wide range of bioinks can be produced to fit specific needs and conditions. By altering the hydrogel concentration, it is possible to alter the mechanical and swelling properties of agarose, thus enhancing versatility of AG for use in tissue engineering. The main drawback of using AG as a matrix/support in cell-laden bioinks is that the hydrogel lacks extracellular adhesion motifs to support cell attachment (Cambria et al., 2020). The use of agarose and its blends as bioink in tissue engineering applications can be found in angiogenesis (Köpf et al., 2016), neurogenesis (Gu et al., 2016), corneal substitutes (Campos et al., 2020), cartilage regeneration (Daly et al., 2016; Salati et al., 2020), cardiac tissue regeneration (Agarwal et al., 2021) and bone regeneration (Kazimierczak et al., 2019). In extrusion-based 3D printing, agarose can also be used as a printing bed to prevent fragile bioink from collapsing prior to crosslinking; i.e., as printed bone scaffolds using nanocomposite bioink in an agarose fluid gel (Cidonio et al., 2019). Furthermore, Adib et al. fabricated a tissue scaffold inside a living patient (intra-corporeal tissue engineering) using agarose support via direct-write 3D printing (Adib et al., 2020).

### 2.4 Gelatin

Gelatin (a partially hydrolysed form of collagen) is a water-soluble, biodegradable polypeptide. The gelling properties of gelatin depend on the source of the material (e.g., mammalian-derived gelatin, fish-derived gelatin). The use of gelatin in tissue engineering applications is limited due to its higher enzymatic degradation rates and poor mechanical and thermal stability owing to high solubility in physiological environments (Hoch et al., 2013). Several studies were done on gelatin-only scaffolds, although the prolonged times and extreme conditions needed for cross-linking lead to limited possibilities for embedding cells (Young et al., 2005; Schuurman et al., 2013). However, gelatin-based hydrogels have been extensively studied in the field of tissue engineering. In such applications, the approach is to incorporate natural and synthetic polymers, as well as other inorganic materials, together with the gelatin in order to increase the stability of the bioink. Notably for cell-laden bioinks, gelatin derived from native collagen contains a RGD cell recognition signal capable of binding to the cell surface receptors. Gelatin is water soluble, biodegradable, noncytotoxic, and nonimmunogenic (Irvine et al., 2015). The formation of gelatin scaffolds happens at low temperatures. However, at physiological temperatures, the viscosity of gelatin drops significantly. Methacrylation of gelatin is a common approach for the fabrication of gelatin scaffolds that can be printed and maintain shape fidelity at physiological temperature (Hoch et al., 2013).

Gelatin methacrylate (GelMA) is obtained from naturally derived polymer gelatin combined with methacrylic groups. Due to its biocompatibility, GelMA is often considered for tissue engineering (Bupphathong et al., 2022). The main properties of GelMA can be controlled in various ways in order to influence interaction between the material and the cells. For example, stiffness and porosity can be controlled by tuning concentration, degree of functionalisation, UV intensity, and additive supplementation (Klotz et al., 2016; Pepelanova et al., 2018). GelMA can be combined with other biomaterials for the development of a broad range of bioinks. For example, it can be combined with hyaluronic acid and mesenchymal stem cells (MSC), to obtain a dermal substitute featuring adipose derived stem cells (ADSC). Such constructs can be used to improve skin regeneration on difficult wound beds by stimulating rapid neovascularization (Eke et al., 2017) or in association with Chitosan for bone regeneration (Suo et al., 2018).

### 2.5 Collagen

Collagen is one of the major components in all the connective tissues, making it one of the most studied biomolecules of the extracellular matrix (ECM), with a triple helix structure. Collagen is the main protein in the ECM of mammalian cells and represents approximately 25% of the total dry weight of mammals (Gelse, Pöschl, and Aigner, 2003; Antoine, Vlachos, and Rylander, 2014). Collagen possesses tissue-matching physicochemical properties and biocompatibility. Even though collagen can be extracted from almost every living animal, the sources for tissue engineering applications include porcine skin, bovine skin and tendons, and rat tail among others. Out of 29 distinct collagen types, only collagen types I, II, III, V, and XI are known for making collagen fibres. Type I collagen (Col-I) is the most investigated for bioprinting uses, due to its ability to undergo self-assembly and form fibrous hydrogels. Because of the slow gelation, structural stability cannot be achieved immediately after bioprinting. At the same time, slow gelation reduces homogeneity in cell distribution, as cells tend to settle near the bottom of the structure due to gravity. So, collagen cannot be used alone without a further support material due to its weaker mechanical properties and slow gelation rates (Lee et al., 2015). Several studies reported the successful incorporation of collagen into a different type of natural and synthetic hydrogel system (Hospodiuk et al., 2017). Bioprinting studies involving the use of collagen have covered the fabrication of skin tissue, muscle tissue and even bone tissue. Applications of collagen are discussed by Yoon et al. (Yoon et al., 2016): 3D skin substitutes were produced using pure (single-component) collagen bioinks: in particular, human epidermal keratinocytes (HEK) and human dermal fibroblasts (HDF) were used to fabricate cell-laden 3D scaffolds through extrusion bioprinting. Produced scaffolds featured four layers: the top one containing keratinocytes and the other three fibroblasts. The effectiveness of the cell-laden 3D scaffolds was demonstrated in a 1 × 1 cm^^2^ full-thickness excision mouse model, with the damaged skin almost completely and clearly regenerated after 1 week. The hair follicles on the wound bed also regenerated almost perfectly. Another possible application of collagen bioinks is in orthopedics. Due to its molecular structure, collagen is used together with other biomaterials and mesenchymal cells for the development of bone-mimicking constructs. In a recent study, the use of a highly porous collagen/hydroxyapatite (HA) scaffold embedded with human adipose-derived mesenchymal stromal cells (hASCs) and human umbilical vein endo thelial cells (HUVEC; EA. hy926) was used for successful spinal fusion in an osteoporotic mouse model. In the cell-laden scaffold, these two typical cell types were used for inducing efficient vasculogenesis and osteo genesis, which can be achieved by several signaling pathways between stem cells and endothelial cells (Yoon et al., 2016).

### 2.6 Decellularized scaffolds

Decellularized materials have been used to obtain scaffolds in various research endeavour involving blood vessels, renal bladders and cardiac valves. The most common material used for scaffold-based tissue engineering is the extracellular matrix (ECM). ECM properties influence cell anchoring, morphogenesis, signaling, and survival. ECM is associated with various structural proteins (collagens, elastin, laminin, and fibronectin), growth factors, and glycans (for example, hyaluronic acid small, leucine-rich proteoglycans (SLRPs), modular proteoglycans, and cell-surface proteoglycans (Frantz, Stewart, and Weaver, 2010). Decellularized ECM (dECM) can be a useful tool for the preparation of bioinks for 3D bioprinting (Abaci and Guvendiren, 2020). The most successful applications of decellularized tissues are for bone and skin, and recently at the organ level. However, dECM limited mechanical properties and fast degradation rate still hampers it use if bioinks for clinical application (Chen et al., 2023). A possible method to improve the mechanical proprieties of dECM involves its combination with natural and synthetic polymers, an approach that gave good results in tissue regeneration (Zhang et al., 2022; Shen et al., 2023; Shi et al., 2023).

One of the main causes hindering tissue healing is over-infection at the site of the wound. The development of dECM-based biomaterials with antibacterial properties could provide a further breakthrough for tissue regeneration. Currently the application of dECM hybrid scaffolds in the form of nanofiber films, sponges, hydrogels, and 3D meshes in regenerative medicine has been continuously improving, in part thanks to the development of new dedicated synthesis techniques such as electrospinning, molding, and 3D printing.

### 2.7 Commentary on biomaterials

Bioinks represent both a great resource and a tricky challenge for researchers and clinicians as their development must consider printability on the one hand and cell viability on the other hand. The currently available literature on tissue regeneration recommends the use of at least two biomaterials in the creation of bioink blend, such as alginate and cellulose nanofibrils. Indeed, currently one of the most investigated materials is alginate, thanks to its rapid gelation when exposed to various ions including Ca^2 +^, Ba^2+^ and Sr^2+^. Independently of the material used, the application of bioinks still faces critical issues that limit its application on a large scale, such as the lack of standardized protocols and legal regulations, as well as the need to fall within the “biofabrication window”, i.e., the need to be suitable for utilization with the chosen biofabrication technology. In general, the choice of material, cellular component and manufacturing methodology are the result of an interdisciplinary dialogue to achieve a customized result for the patient.

## 3 3D printers for extrusion-based bioprinting

The interest in extrusion-based bioprinting has steadily increased in recent years. Forecasts made for the years ranging from 2021 to 2028 call for a yearly growth rate of the bioprinting market of 15.8% per year (Grand View Research, 2020). Despite the increasing interest in the subject, commercial solutions in terms of bioprinting machines still have prohibitive costs, especially if compared with the prices of standard extrusion-based printers. For this reason, in the literature, along with commercial solutions, it is often possible to find a wide variety of bioprinters created starting from scratch or modified starting from commercial, material extrusion machines not created for bioprinting. The second solution seems to be preferred, as it allows to contain the costs of developing a bioprinting machine by modifying reliable existing architectures with all the advantages of having a commercial framework. Another major point of differentiation between commercial and in-house built solutions is flexibility to accommodate innovative research. Commercial solutions are often black-boxes when considering access to internal functionality, and provide limited degrees of freedom to researchers wanting to explore bioprinting and applications beyond the realm of intended usage scenarios. On the contrary, custom solutions allow increased control on the internals of hardware and software, giving more flexibility. A typical case in point is research on multi-material bioprinting, where there is usually a strong need to modify printing strategies beyond what is possible when using commercial solutions. On the other hand, choosing a commercial bioprinting system usually often means having state-of-the-art printing solutions ready for use, with significant time saved if the research focuses on innovative products, rather than innovative materials or processes. However, this latter point is questionable in that real innovation often comes as a combination of product, material and process.

### 3.1 Commercial MEX-based bioprinters

Commercial bioprinting machines based on material extrusion are characterized by high accuracy in material deposition, often enhanced by the use of pneumatic dispensing systems, chosen by the majority of the producers. Most of bioprinter manufacturers currently opt for modular systems, in which extruders can be conveniently replaced in order to provide different deposition technologies (e.g., thermoplastic material extrusion or a droplet deposition system (CELLINK, 2020)). Some systems feature multiple deposition heads mounted on the machine at the same time. In some cases, it is possible to replace one of the extruders with UV systems for curing (Regemat3D 2016), (Advanced Solutions, 2015), or with monitoring systems (Advanced Solutions, 2015). Some manufacturers of commercial bioprinting systems also provide a heterogeneous portfolio of machines to accommodate different use-cases, for example, distinguishing between machines for end-users and machines for developers, the latter typically implemented to favour hardware modifications for research purposes (Desktop Health, 2013; CELLINK, 2015). An overview of the main commercial extrusion-based bioprinter models is presented in Table 1.

**TABLE 1:** General view of commercial extrusion-based bioprinters. The table summarises the main features and applications for each machine: company, extrusion technology, presence of curing and heating systems, number of extruders (including fused filament fabrication extruders), possible applications, starting price and release year.

Machine commercial name	Company	Extrusion system	UV curing	Heated extruder	Heated bed	n. extruder	Other technologies	FFF	Applications	Price (starting from)	Year (first release)
Allevi3	Allevi by 3D systems (Allevi, 2018)	pneumatic	x	x	x	3		no	heterogeneous tissues	$40000	2018
Bio v1	Regemat 3D (Regemat3D 2016)	piston-based	x	x	x	3	max 3 extruders replaceable with other systems	yes	heterogeneous tissues	$25000	2016
3D-Bioplotter Developer Series	Desktop health (Desktop Health, 2013)	pneumatic	x	x	x	max 3		no	heterogeneous tissues	$100000	2013
Bio x	Cellink (CELLINK, 2020)	pneumatic	x	x	x	3	replaceable 4 different tech (e.g., FFF, droplets)	yes	heterogeneous tissues + vascularisation	$39000	2020
inkredible	Cellink (CELLINK, 2015)	pneumatic	x			2		no	heterogeneous tissues	$5000	2015
Dr. INVIVO 4D	Rokit (ROKIT Healthcare Inc, 2016)	pneumatic			x	5 + 1 interchangeable	chamber temperature control + built in optical microscope	yes	heterogeneous tissues	$50000	2016
Bio Assembly Bot 400	Advanced Solutions Life Sciences (Advanced Solutions, 2015)	pneumatic and/or piston-based	x	x	x	up to 8	Camera + robotic arm	no	organoids	$100000	2015

### 3.2 Custom MEX-based bioprinting machines

Custom solutions are frequently adopted in bioprinting applications based on material extrusion. All the custom solutions summarised in the following Table 2 rely on a Cartesian architecture, both when the machine is designed from scratch and when it is developed starting from a commercial frame. In some cases, when starting from a commercial machine, developers choose to maintain also the original extrusion system (typically designed for fused filament fabrication–FFF: a process where a continuous filament is melted and extruded through a nozzle), as the original extrusion system is still useful to create non-bioink support structures for the subsequent bioink deposition (Goldstein et al., 2016; Krige et al., 2021). In other cases, bioprinters have been developed starting from frames of computerized numerically controlled (CNC) machine tools, i.e., machines originally designed to perform material removal tasks. In such cases, the adaptation typically consists of replacing the material removal head with one or more material extrusion heads, often accommodating different extrusion technologies (Yenilmez et al., 2019). Concerning the extrusion technologies, while in commercial machines the pneumatic solution for material dispensing is the most popular, most of the custom machines developed for research purposes exploit piston-based systems. The main reason for privileging the piston-based system is that most of the custom solutions are based on commercial FFF machines modified by replacing the extrusion system and the installation of a pneumatic system would require also a pressurised solution, i.e., handling more components and a more complex system (Goldstein et al., 2016; Roehm and Madihally, 2018; Bessler et al., 2019; Kahl et al., 2019; Ioannidis et al., 2020; Krige et al., 2021).

**TABLE 2:** General view of custom extrusion-based bioprinters. The table summarises the main features and applications for each machine: company, extrusion technology, presence of curing and heating systems, number of extruders (including fused filament fabrication extruders), possible applications, starting commercial/custom frame, starting price and release year.

Company	Extrusion system	UV curing	Heated extruder	Heated bed	n. extruder	Other technologies	FFF	Applications	Starting architecture	Price	year
University of Patras, Patras, Greece (Ioannidis et al., 2020)	piston-based				1		no	heterogeneous tissues, bone	3D Anet A8	$230.00	2020
Jagiellonian University, Cracow, Poland (Mielczarek et al., 2015)	piston-based	x	x		1		no	heterogeneous tissues	custom	Non- available	2015
Oklahoma State University, United States (Roehm and Madihally, 2018)	piston-based			x	1		no	Cell-laden structures	Maker’s Tool Works	$1000	2017
University of Iowa, United States (Ozbolat, Chen, and Yu, 2014; Moncal et al., 2019)	pneumatic		x	x	2	double extruder, one for the scaffold, one for cell spheroids	no	bone, cartilage	custom	Non- available	2019
Friedrich-Alexander-University Erlangen-Nürnberg, Germany (Kahl et al., 2019)	piston-based				1		no	kidney, cell-laden geometries	Anet A8	$150–200	2019
University of Connecticut, United States (Yenilmez et al., 2019)	piston-based + pneumatic for droplets	x			2	Droplet and material extrusion	no	heterogeneous tissues	CNC stage (2020B, Konmison, China)	$1370	2019
The Feinstein Institute for Medical Research, New York & Hofstra Northwell School of Medicine, New York. (Goldstein et al., 2016)	piston-based				2		yes	cartilage	MakerBot Replicator 2X Experimental 3D Printer	∼300$	2016
Ruhr University Bochum, Germany & University Medical Center Utrecht, Netherlands (Bessler et al., 2019)	piston-based			x	1	Dedicated custom software	no	human kidney cells, mouse embryonic stem cells	Prusa i3	commercial platform +$80	2019
Luleå University of Technology, Sweden (Krige et al., 2021)	piston-based	x			2		yes	bacteria, cell-laden geometries	Prusa i3	commercial platform +300$	2021
University of Toronto, Canada (Fitzsimmons et al., 2018)	piston-based		x		5		no	Human umbilical vein endothelial cells, adipose-derived stromal cells	custom	$3000	2018

## 4 Process variables and control strategies for extrusion-based bioprinting

**FIGURE 3:**
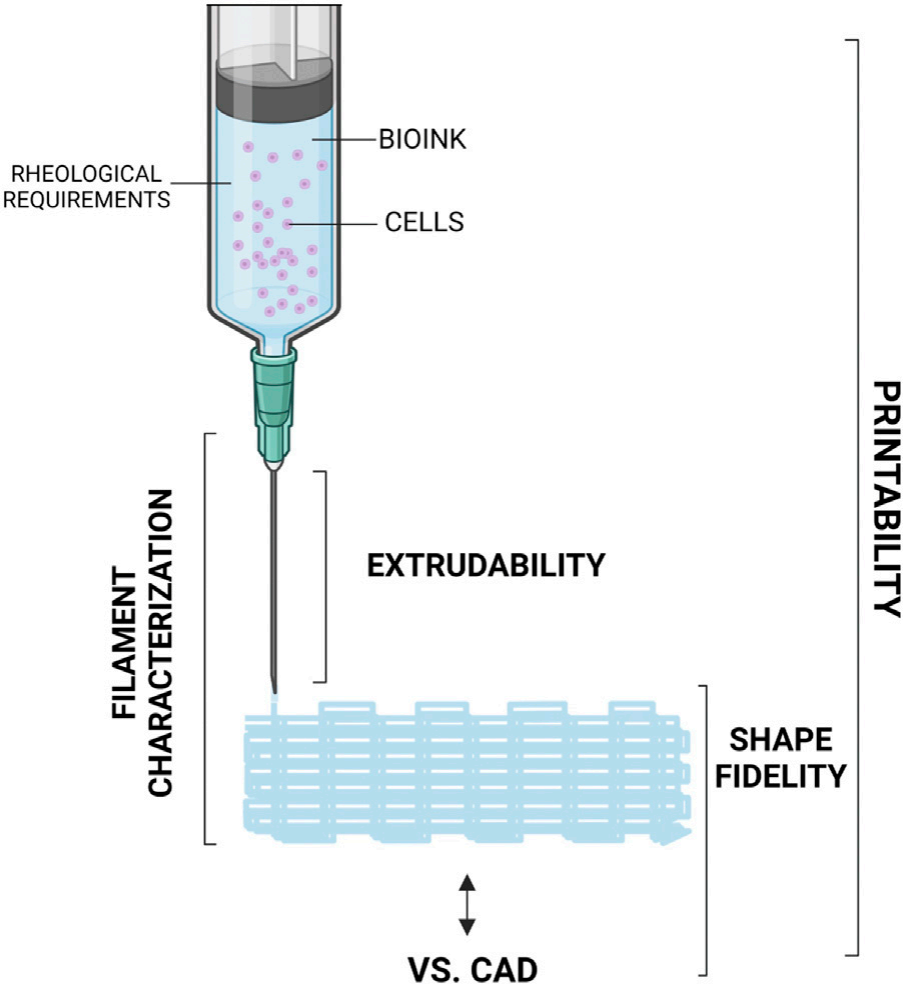
Schematisation of an extruder for 3D bioprinting of bioink. Created with BioRender.com.

The term “printability” is used in extrusion-based bioprinting to indicate if a specific bioink can be successfully extruded and deposited to build a certain part, and if the final construct possesses the mechanical and biological properties that were required by design (see Figure 3), where biological properties relate mainly to viability (i.e., survival in healthy status) of the embedded cells. For any given bioink, printability is generally guaranteed under the assumption that extrusion is performed under a well-specified, constrained set of operating conditions. The choice of extrusion process parameters is critical to determine whether a bioink is considered suitable for extrusion-process bioprinting. Moreover, most of the process parameters are reciprocally affected, making it possible to extrude the same material by combining them in different ways (Mahadik et al., 2023). Here we present an overview of the most influential process variables and how they affect the process. For a recent paper that proposes an original method to identify optimal combinations of process parameters in order to guarantee higher cell viability as well as geometrical fidelity, see (Zanderigo et al., 2023). As the gradual development of 3D bioprinting led to the possibility of incorporating a huge range of biomaterials with cells and biomolecules for *in vitro* tissue generation, the production of an open-source database was necessary. With this aim, Mahadik et al. created an openly available repository which allows to researchers to immediately find the already tested 3D printing formulations (Center for Engineering Complex Tissues, 2022).

### 4.1 Extrusion variables

#### 4.1.1 Pressure

In order to allow the material to be extruded, three solutions are currently adopted in most of the extrusion-based bioprinting systems, to transmit the necessary amount of pressure: 1) pneumatic, 2) screw-based and 3) piston-based systems. In the second and third system, force is applied via mechanical contact, while in pneumatic systems compressed air is used to overcome the surface tension of the bioink and force it through the nozzle. The higher the yield stress of the material, the more pressure is needed to initiate and maintain the extrusion. Another element of consideration when choosing extrusion pressure is cell viability: cells react negatively to increasing amounts of pressure to withstand, in particular when pressure translates into shear-rate for cells closer to the walls of the extrusion chamber, as discussed later in this section. Usually, a compromise must be made between applying enough pressure to guarantee the extrusion, but not too much to avoid damaging the living cells. Among the three aforementioned solutions to implement extrusion, because of the specific flow patterns they generate, screw-based extrusion systems were proven to induce more damage to the embedded cells (Ning et al., 2020). Despite that, screw-based systems are still preferable when using high-viscosity bioinks because of their intrinsic capability to generate higher pressures needed to initiate the extrusion (Hölzl et al., 2016). Larger pressures are also often desired when the aim is to obtain high accuracy in the printed part, thanks to more accurate dosing and compacting of the material (Hölzl et al., 2016; Ning et al., 2020).

Depending on the applied pressure, hydrogels with different gel content react differently in terms of deposition performances (B. K. Wang et al., 2022). Pressure is therefore a key factor to guarantee printability of the bioink. Note that applying more pressure than required may result into unstable material flow, whilst too-low pressures may cause discontinuity in deposited material (T. Gao et al., 2018). The challenge with pressure is that it can hardly be directly controlled within the entire volume of bioink being subjected to the extrusion process. Pressure is applied on the bioink surface by external means, but local pressures felt internally by the bioink will vary across the extrusion chamber, depending on local traversal speeds within the chamber, and on local chamber geometry. The combination of diferent values of pressure and nozzle diameter can indeed lead to different printing conditions, as proved in (Mahadik et al., 2023) for Poly (ε-caprolactone) (PCL) and GelMA. Material viscosity may also change along the extrusion chamber leading to different effects of the same applied pressure felt by different regions within the material. In (Paxton et al., 2017), the authors implemented a mathematical model to determine the correlation between pressure, extrusion velocity and nozzle geometry. Other models were focused on correlating pressure with storage modulus and loss modulus, creating a methodology to determine the optimal pressure to be applied to the bioink, knowing its properties, to ensure printability (Gao et al., 2018).

As stated earlier, a key aspect to be considered when determining the extrusion pressure is cell viability. In combination with nozzle geometry, pressure is indeed one of the two most influential parameters for cell survival rate, as it is responsible for the shear stress to which cells are subjected during the extrusion process (Gao et al., 2018; Paxton et al., 2017). In (Nair et al., 2009), it was investigated how the percentage of cell viability can change dramatically exclusively due to the change in pressure, measuring a variation in survival rate up to 38.75%.

#### 4.1.2 Temperature

As high temperature is the first cause for material degradation, temperature must be controlled during and after the extrusion to ensure higher cell survival rate (Giuseppe et al., 2018). After the material exits the extruder, temperature can be exploited as a means to control the crosslinking process when using thermo-responsive gelatins (Moncal et al., 2019; Gillispie et al., 2020). Low temperatures usually slow down the cross-linking process, allowing for the formation of a more ordered network and a more stable final structure in alginate gels (Drury, Dennis, and Mooney, 2004; Roehm and Madihally, 2018). Other materials (e.g., Pluronic^®^ F127) may behave in the opposite way, creating stable structures with the increase of temperature. These latter materials are usually more apt to function as scaffolds in bioprinting (Moncal et al., 2019).

During the deposition, also the temperature of the build plate plays a relevant role in printing accuracy and in obtaining the desired properties in the deposited material (Roehm and Madihally, 2018; Koch et al., 2020). Temperature control can be applied to the build plate, for example, by using a cooling system to chill the deposited material, with the aim of improving the stability of the printed structure (Rasheed et al., 2021).

The effects of temperature have been investigated also during extrusion, demonstrating influence on material viscosity and as a means to enhance bioink printability (Billiet et al., 2014; Roehm and Madihally, 2018; Moncal et al., 2019). In (Wang et al., 2022), various temperatures (37°C, 45°C and 53°C) were tested to investigate how viscosity changes and influence on shear rates.

Temperatures are also crucial when considering cell viability (Roehm and Madihally, 2018). Solutions were proposed to maintain a constant temperature of 37°C at the nozzle tip (Moncal et al., 2019) and for the entire batch of material to be printed (Fitzsimmons et al., 2018). By controlling both needle and chamber temperature it is possible to have an increase in cell viability up to 35% (Ouyang et al., 2015).

#### 4.1.3 Nozzle, orifice or needle geometry

Extrusion-based bioprinting makes use of either conical or cylindrical outlets. The choice depends on material properties and application (desired results), influencing also the choice of printing parameters. The outlet geometry is particularly important in determining the shear stress felt by the living cells, because it features the narrowest cross-section the material has to pass through, thus greatly affecting cell viability (Billiet et al., 2014).

Comparisons between cylindrical nozzles and conical ones demonstrated through finite element simulation that, even if conical outlets result in lower average values of shear stress, the stress at the exit side is one order of magnitude higher than the maximum value for the cylindrical outlet (Townsend et al., 2019). Nevertheless, even if maximum shear stress is higher, conical needles are preferable to enhancing cell viability in specific situations: 1) when the applied pressure is lower than 1 bar (for higher pressures a significant drop in the survival rate was observed (Townsend et al., 2019)). and 2) when the extrusion process is characterised by a high velocity (Ates and Bartolo, 2023). Elsewhere, it was proven that under equal conditions conical nozzles enhance the possibility of cell survival in the majority of the cases (Lucas et al., 2021).

**FIGURE 4:**
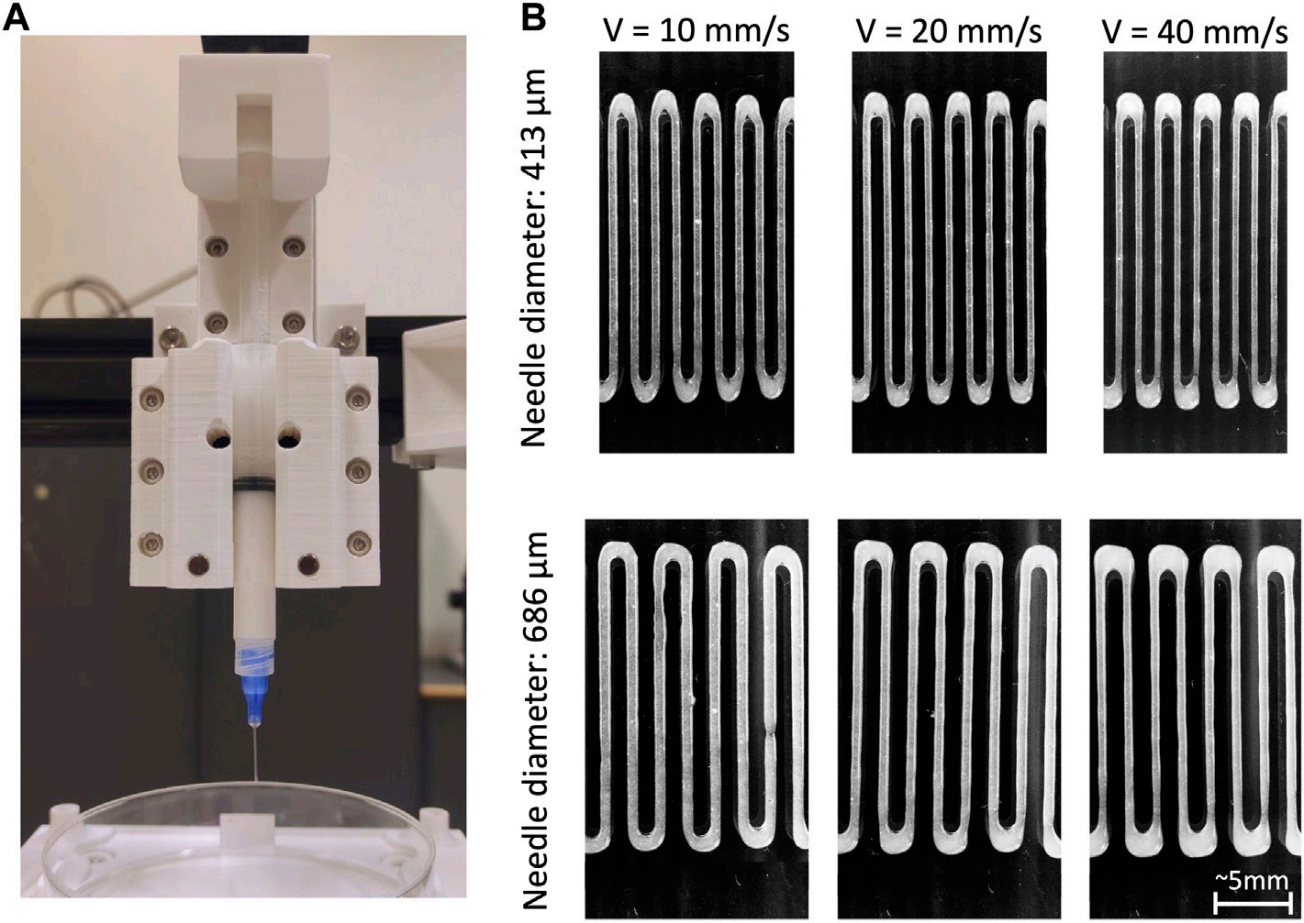
**(A)** Example of a piston-based extrusion system; **(B)** strand of bioink (microfibrillar cellulose 1.5%) deposited by the piston-based system using increasing printing speeds (10 mm/s, 20 mm/s, 40 mm/s) and two different nozzle diameters (413 μm and 686 μm). Other process parameters were kept constant.

The internal diameter of the extrusion outlet plays an important role in extrusion-based bioprinting. A larger diameter increases cell survival rate, however it causes a decrease in printing accuracy (Chand, Muhire, and Vijayavenkataraman, 2022). Conversely, smaller diameters allow for higher resolution in deposition (see Figure 4), but more often lead to clogging and to increased extrusion pressure, with a consequent increase in distress for cells (Malekpour and Chen, 2022). Moreover, the increased stress experienced during the extrusion may also affect the viability in the days following the printing process (Townsend et al., 2019). The correlation between nozzle diameter and cell viability was extensively investigated, showing that for cylindrical needle the cell viability improved by ∼7% when increasing the diameter from 160 μm to 260 μm, while no significant differences were found between diameters from 260 μm to 510 μm (Ouyang et al., 2015). Concerning conical needles, computational fluid dynamics (CFD) models and experimental results demonstrated that, even when using different outlet diameters (from 250 μm to 840 μm), there is no appreciable difference in cell viability (Emmermacher et al., 2020).

Shear rate has proven fundamental at influencing not only cell viability, but also cell function. In general, cell damage, and thus potential alteration of functionality, grows exponentially with the increase of shear stress (Boularaoui et al., 2020). Although cells are often capable of self-recovering after experiencing large deformations and shear stress (up until a certain limit) (Secomb, 1991), in most cases, cells are likely to report damages to the membrane after the extrusion (Giuseppe et al., 2018; Ning et al., 2018). Depending on the stress magnitude, cells may undergo different deaths, i.e., lysis, necrosis, and apoptosis, or change their phenotype (Ning et al., 2018; Lemarié et al., 2021). In other cases, cells were reported to have abnormal behaviour in proliferation and differentiation after the extrusion, with a consequent reduction in the capability of creating functional tissues through bioprinting (Ning et al., 2018; Boularaoui et al., 2020).

#### 4.1.4 Flow rate and traversal speed

Both material flow rate through the extrusion orifice and nozzle traversal speed over the build plate provide information about the overall speed of the printing process. Flow rate, also referred to as “dispensing velocity”, indicates how fast the material is forced to exit through the nozzle, while traversal speed refers to the printer movements in *x* and *y* directions, i.e., the velocity of the extrusion head whilst moving along the predetermined deposition path.

Flow rate is directly linked to applied pressure, as the more pressure is applied, the faster the material goes through the nozzle (Chand, Muhire, and Vijayavenkataraman, 2022). Flow rate also directly affects residence time, i.e., the time during which cells are subjected to high pressure inside the extrusion chamber before exiting the nozzle, which in turn affects their viability. A longer residence time is indeed associated with a decrease in cell survival rate. Cells travelling closer to the walls of the extruder have fewer chances of survival (Paxton et al., 2017). This is due to the parabolic velocity profile from the walls to the centreline of the extruder, which allows the cells in the centre to move quickly towards the exit (Snyder et al., 2015). Ideally, having a higher volumetric flow allows for a shorter residence time and thus for an increase of cell viability, though the flow has an upper bound related to fluid viscosity and nozzle geometry (Chand, Muhire, and Vijayavenkataraman, 2022). At the same time, an ideal value for flow rate has to be determined within a small range, as higher flow leads also to an increase in pressure, thus in shear stress (Chand, Muhire, and Vijayavenkataraman, 2022).

The ratio between nozzle traversal speed on the printing plane, and flow rate of the material exiting the nozzle is fundamental to ensure printability of a bioink (Jin, Chai, and Huang, 2017). Combined effects of extrusion feed rate and traversal speed determine the diameter of the deposited strand, as shown in Figure 4. That is, if extrudate flow rate is higher than traversal speed, the cross-section of the deposited strand is larger than the needle aperture, conversely, when flow rate is lower, the cross-section is smaller (Aliabouzar et al., 2022). Consistently, the more pressure is applied in the extrusion, the larger the diameter of the deposited strand (Rastin et al., 2020). Traversal speed directly influences deposition accuracy (Rastin et al., 2020). To achieve continuity and homogeneity in the deposited strands, nozzle traversal speed is a key parameter (Chen et al., 2019): when the nozzle is too slow with respect to the dispensing velocity, strand swelling is generated. Conversely if the nozzle travels too fast, excess speed eventually leads to the strand breaking up into segments or droplets (Jin, Chai, and Huang, 2017).

### 4.2 On-machine sensing and control strategies

On-machine sensing refers to equipping a bioprinter with sensors (mounted onboard) that can be used to observe the conditions of the machine over time, and/or the conditions of the part being made. On-machine sensing solutions presented in the bioprinting literature so far have focused on observing the part in terms of geometrical accuracy and structural integrity, or on observing the cells within the part, to understand their spatial distribution post-print, their survival rate, and their functionality. For observing geometric/structural properties, laser scanning technologies have been applied to measures geometry deviations from the ideal part and to control process parameters to reduce errors (Armstrong, Alleyne, and Wagoner Johnson, 2020; Armstrong et al., 2020; Armstrong et al., 2021). Other geometry inspection strategies were explored using optical coherence tomography with the possibility of receiving feedback during the fabrication (Yang et al., 2021), (Yang et al., 2023). In (Gerdes et al., 2020), the authors focused on guaranteeing structure integrity using an image-based approach, while in (Gugliandolo et al., 2022), images obtained from a coaxial camera were used to evaluate geometrical defects. Machine learning algorithms were also explored to build a monitoring system capable of detecting printing issue through the classification of images collected using a webcam (Bonatti et al., 2022).

As stated earlier, another aspect of on-machine sensing for bioprinting is related to observing cells localisation and viability during print or post-print. In (Poologasundarampillai et al., 2021), hydrogel and suspended cells were observed during the process with light-sheet fluorescence microscopy to evaluate which conditions may lead to cell damage.

## 5 Main applications of extrusion-based bioprinting for tissue and organ fabrication

Progress in bioprinting of several human cells/tissues has been relevant. The human body is comprised of several organs that work and function synergistically to maintain physiological homeostasis. A body system includes, in turn, several organs and tissues aimed at fulfilling a definite action. In the literature, the benefits of bioprinting have been illustrated for different organs/systems such as bones, muscles, nervous, lymphatic, endocrine, reproductive, respiratory, digestive, urinary, circulatory and skin (Vijayavenkataraman et al., 2018). This section illustrates bioprinting applications in models and regenerative medicine.

### 5.1 Bone

Bone tissue bioprinting has gained attention as a viable option to replace damaged or lost bone tissue, compared to traditional approaches consisting of allogeneic implants or transplants (Singh et al., 2018). Bone tissue bioprinting circumvents the risk of unavailability of bone donors for transplant, morbidity at the level of the graft, and risk for transmissible diseases associated with traditional bone graft (Li et al., 2015). For instance, Lee et al. have studied the *in vitro* differentiation of pre-osteoblasts (MC3T3-E1) and *in vivo* bone tissue regeneration at the level of skull defects in rats, upon implant of 3D scaffolds incorporating PLGA microspheres loaded with BMP-2. The scaffolds loaded with BMP-2, printed with micro-SLA technology were associated with high expression of ALP and osteocalcin with improved cell differentiation capacity (Lee et al., 2011). This work has heralded the capacity to embody a bioactive molecule like BMP-2 in a 3D printed scaffold. Furthermore, Temple et al. have worked on a PCL scaffold with variable porosity to form mandible and maxillary bones by using material extrusion. The resulting scaffold has been tested *in vitro* and *in vivo* to assess the ability of adipose tissue-derived stem cells (hASCs) to form bone and capillary tissues within an optimised differentiation process (Temple et al., 2014). Byambaa et al. succeeded in forming 3D bone tissue-like constructs (containing separate osteogenic and vascular niches), by employing extrusion-based direct-writing bioprinting (Byambaa et al., 2017). For this purpose, the authors used two types of bioinks based on GelMa hydrogels (methacrylate). A central vascular fibre has been printed by using rapidly degradable GelMA bioink (GelMA conjugated with 5% VEGF with low methacrylate content (GelMALOW-VEGF) containing HUVEC and hMSCs. Silica-based nano-plates loaded with GelMA bioink (10% GelMAHIGH + VEGF at grading concen-trations) were printed around the central fibre rod to initiate osteogenesis. HUVEC and hMSC, upon co-culture in the bioprinted fibres, retained viability and allowed for cell proliferation and neo-vessel generation. Within a vascularised bone construct, containing a mineral-enriched ECM, osteoblastic maturation, the strong expression of OCN, RUNX2 and CD31 were compatible with the formation and maturation of a bone construct, supported by central angiogenesis, upon 21 days of culture. Such an approach of bone bioprinting, with the help of cells growth, addressed the repair of big bone defects (Byambaa et al., 2017). With the aim to develop modified bioinks associated with optimised biological and physical chemical properties, Ojansivu and others (Ojansivu et al., 2019) have used wood-derived nanocellulose (CNF) and bioactive glass (BaG) to strengthen jelly-alginate bioinks for bioprinting of bone cells (using a commercially available bioprinter, 3D-Bioblotter^®^). In particular, CNF greatly improved hydrogel flows for bioprinting. The viability retention of Saos-2, a human osteoblastic, osteogenic sarcoma-derived cell line, has confirmed the compatibility of the modified hydrogels. Saos-2 cells have retained the viability in BaG-deprived gels, while their viability and proliferation activities declined in the presence of BaG with increased viscosity. These observations significantly improved progress of 3D bone bioprinting by generation of a multi-functional bioink for bone tissue regenerative engineering. In particular, the importance of the viscosity with respect to bioprinting, in terms of regulation of short- and long-term cell viability and pro-liberation has clearly emerged (Ojansivu et al., 2019).

A few clinical studies involving bioprinted bone constructs have been reported so far (Uzeda et al., 2017). Biphase calcium phosphate (BCP) scaffold fabrication was tested in clinical trials (Beheshtizadeh et al., 2022). Mangano et al. (Mangano et al., 2013) reported the case study of a patient undergoing maxillary buccal plate bone regeneration using a 3D-printed construct. Due to the high biocompatibility of BCP, the scaffold was easily integrated into the formed bone area. The 3D printed BCP scaffold was later evaluated clinically and histologically (Mangano et al., 2021). It was found that, after a time period of 7 years, biomaterial volume reduced about 23% and more than 57% of the entire mineralized tissue consisted of newly produced bone tissue. BCP scaffolds in bone substitutes have been the subject of further studies (Sun and Yang, 2015), (Wang and Yeung, 2017). The use of porous ceramic bone scaffolds, used for the reconstruction of bone tissue, has been reported as well (Lin et al., 2019). In a recent study, Poly (ε-caprolactone) (PCL), gelatin (GEL), bacterial cellulose (BC), and different hydroxyapatite (HA) concentrations were used to fabricate a novel PCL/GEL/BC/HA composite scaffold using 3D printing method for bone tissue engineering applications. The addition of both bacterial cellulose (BC) and hydroxyapatite (HA) into a PCL/GEL scaffold increased cell proliferation and attachment. PCL/GEL/BC/HA composite scaffolds provide a possible new approach to bone tissue engineering applications (Cakmak et al., 2020).

In a different study, a hybrid system consisting of 3D printed PCL filled with hydrogel was developed as an application for reconstruction of long bone defects, which are intrinsically challenging to repair as large segments of the bone may be entirely missing. The hydrogel was a mixture of alginate, gelatin, and nano-hydroxyapatite, infiltrated with human mesenchymal stem cells (hMSC) to enhance the osteoconductivity and biocompatibility of the system (Hernandez, Kumar, and Joddar, 2017).

### 5.2 Cartilage

**FIGURE 5:**
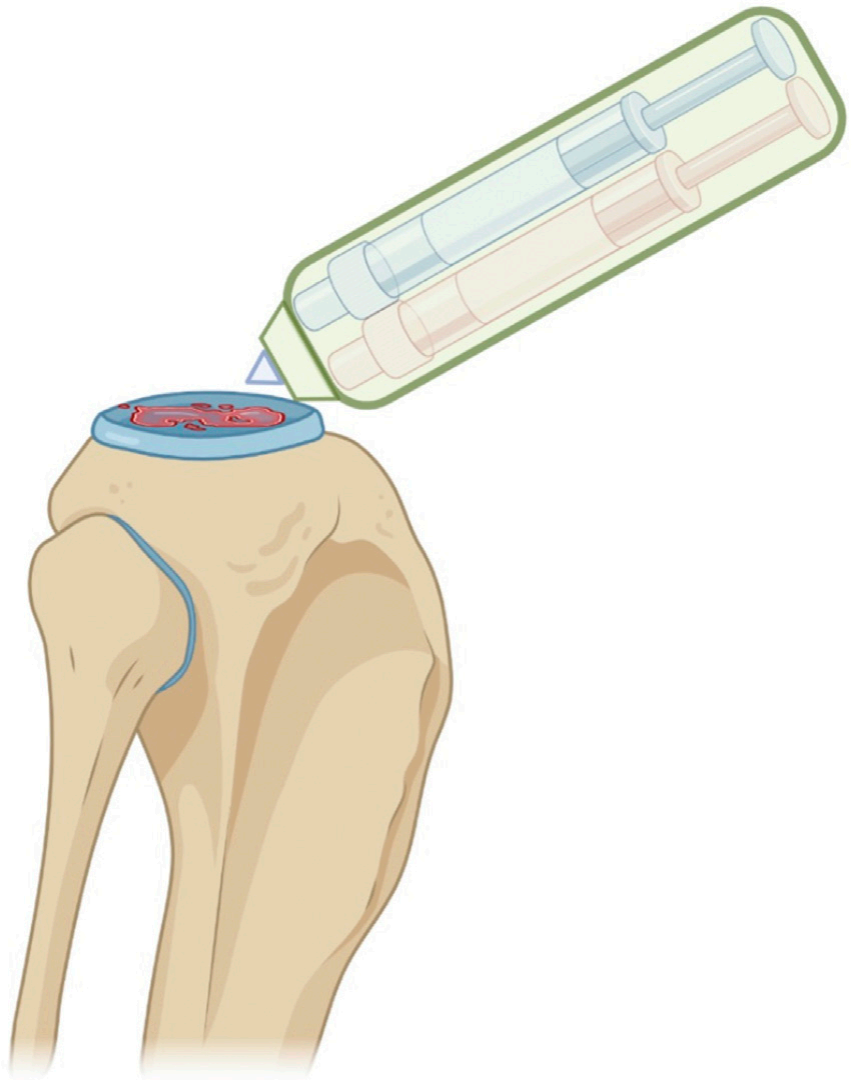
Schematic representation of the *in situ* handheld bioprinting technology used by Bella et al. for cartilage regeneration (Bella et al., 2018). Created with BioRender.com.

Yang et al. (Yang et al., 2018), used collagen/alginate bioinks for cartilage regeneration. Jiang et al. (Jiang et al., 2019), reported that chondrocytes show elevated cell viability and good proliferation with highly polymerized actin. In the study, the expression of internal glycosaminoglycans was increased over time and cartilage-specific markers were upregulated in the 3D printed structure. Markstedt et al. printed a cell-laden construct for cartilage regeneration, demonstrating high shape fidelity and size stability, together with good cell distribution and >70% viability (Markstedt et al., 2015). O 'Connell et al. developed a new portable device, or “Biopen”, that allows use of bioprinting and manual control of scaffold deposition during surgery, with or without live cells (O’Connell et al., 2016). The Biopen is basically a portable extruder based on a pneumatic dispensing system and equipped with a photocuring source. It was employed by the Group to create GelMA/HAM bioscaffolds, consisting of a core and a shell and have a mechanical resistance of 200 Kpa and retention on encased cell viability for chondral tissue repair. The work from O'Connell et al. unfolded the great potential of multi-ink bioprinting, with special regard to co-axial bioprinting for *in vivo* application, also during surgery. The portable Biopen has also been used to study deep chondral defects in sheep animal models and proved the safety and the potential clinical efficacy of the system. This has represented the first approach to an *in-situ* 3D bioprinting, holding a great potential for future clinical application (O’Connell et al., 2016; Duchi et al., 2017; Bella et al., 2018) (Figure 5). Rathan et al. have developed alginate bioinks, similar to the cartilage ECM (cECM) for the fabrication of cartilage tissue. These bioinks could improve MSC viability upon bioprinting, as well as *in vitro* chondrogenesis. Bioinks with high cECM content have been associated with higher gene expression of COL2 and ACAN (Aggrecan). These bioinks supplemented with MSC and TGF-β3 have resulted in a robust chondrogenesis, which can make them suitable for direct cartilage tissue repair (so called print and implant). The study has introduced a new class of functionalised bioinks that may apply to regenerative engineering of muscle-skeletal system (Rathan et al., 2019).

In a recent study Tang et al. explored the use of 3D printing technology to fabricate bioactive artificial auricular cartilage using chondrocyte-laden gelatin methacrylate (GelMA) and polylactic acid (PLA) for auricle reconstruction. In this study, chondrocytes were loaded within GelMA hydrogel and combined with the 3D-printed PLA scaffolds to biomimetic the biological mechanical properties and personalized shape (Tang et al., 2021).

### 5.3 Liver

The applications of 3D printing applied to the liver are diverse. Firstly, many drugs exhibit hepatotoxicity or are metabolized in the liver. The opportunity of 3D printing in drug screening and the study of pharmacokinetic parameters becomes highly relevant. Secondly, it enables the creation of physiopathological models without the need for small animals. Furthermore, this technique could be employed for personalized medicine or regenerative medicine. Interestingly, within the regenerative medicine field, the possibility to bioprint different cell types has been exploited to fabricate more complex organs. Lee et al. have bioprinted primary rat hepatocytes (HCs) together with HUVEC’s and human pulmonary fibroblast (HLF) using multiple heads to bioprint liver tissue. The 3D constructs have been obtained by delivering cells containing collagen bioinks into poly-caprolactone-based canaliculi structures. The co-culture 3D microenvironment has resulted in heterotypic interaction between the cells with an improvement of HC viability and function, in terms of albumin and urea synthesis by the bioprinted liver prototype. In summary 3D bioprinted constructs containing capillary networks proved to be useful for functional liver tissue regeneration (Lee et al., 2016). Cuvellier et al. (Cuvellier et al., 2021) have first bioprinted hepatic cell line (HepaRG) together with hepatic parenchymal cells, stellate cells (LX-2) an dHUVECs. The 3D construct, when exposed to TGFβ-1, exhibited the presence of fibrillary collagen deposition necessary for fibrogenesis. In a subsequent study (Cuvellier et al., 2022), they bioprinted primary human hepatocytes within a methacrylated gelatin matrix. These hepatocytes remained polarized, viable for 28 days, and demonstrated activities of phase I and II biotransformation enzymes. Upon implantation in mice, these 3D constructs demonstrated their ability to vascularize and secrete albumin.

### 5.4 Nervous tissue

Another interesting field of application for bioprinting regards the development of new scaffolds for further improvements. Lozano et al. generated a hand-held extruder for the preparation of 3D multi-layered structures, similar to the brain. The bioink, made of arginylglycylaspartic acid (RGD) induces cells adhesion to ECM: ECM conjugated with modified gellan (RGD-GG) embodies primary neuronal cortical cells, making a cortex tissue. The developed bioink is able to support cell viability (>70%), regardless of the employed cross-linker (i.e., DMEM or CaCl2), and is suitable for the formation of a cell network. Cortex neural cells responded better to RGD-GG than GG purified hydrogels, showing higher viability. The work greatly contributed to the study of neural network damage after trauma through the fabrication of multi-layer structures containing cells to form a complex 3D model *in vitro* (Lozano et al., 2015). Kilian et al. (Kilian et al., 2022), bioprinted brain tissue using a combination of alginate and agarose to create a multi-zonal anthropomorphic MRI phantom. This phantom was developed specifically to study MR relaxation times T1 and T2.

3D bioprinting technology in neurologic disorders addresses also the development of solutions to repair peripheral nerves lesions. A bioink comprised of sodium alginate, carboxymethyl chitosan and agarose, and containing neural stem cells (NSC), allowed to obtain cell differentiation towards formation of neural synapses and network connections. Interestingly, these cells were able to secrete Calcium in response to the stimulus of bicuculline, typical of neural cells. Recently, Ning et al. fabricated 3D scaffolds containing bioink with Schwann cells (SC), using low-viscosity polymers based on modified alginate hydrogel, RGD, HA and fibrin. Gel composition and the printing process have been optimised to retain high SC viability (90%) after bioprinting (SCs>90%). Furthermore, SC morphology and neural cell growth could be changed by modifying printing velocity. This work has shown feasibility of bioprinting of 3D scaffolds with low-viscosity bioinks, hence enabling migration of SC for peripheral neural tissue repair (Ning et al., 2019).

### 5.5 Skin

The skin is the largest organ in the body, comprising a multi-layered structure that protects muscles, bones, ligaments, and the underneath organs. It represents the first line of defence against external solicitations, and being the most vulnerable to lesions, it requires regeneration strategies that are quick and reliable. Skin substitutes by tissue engineering are intended to overcome actual limits of the traditional treatments for skin diseases in terms of technology, time and costs. Furthermore, the cosmetic industry is also highly interested in 3D skin models for conducting tests while avoiding the use of animals. In spite of the accrued progress, for the treatment of wounds and superficial lesions, the management of deep wounds, with special regard to third/fourth degree burns is far from applicability.

Approximately 61% of skin-related constructs manufactured by material extrusion lack complexity and feature a single cell phenotype, which mainly consist of dermal fibroblast (Osidak et al., 2019; Won et al., 2019), (Shi, Laude, and Yeong, 2017; Dubbin et al., 2016; Tigner et al., 2020). These studies can be considered as a starting point towards the creation of actual 3D-bioprinted skin constructs (Perez-Valle, Amo, and Andia, 2020), though most of the printable structures are not yet capable to represent all the functions of skin tissue. Printed structures integrating multiple cell phenotypes, with complex molecular crosstalk, are required. The recovery of skin homeostasis requires the interaction between fibroblasts and keratinocytes, to allow fibroblasts to produce tissue-forming factors such as the keratinocyte growth factor (KGF) and fibroblast growth factor (FGF) (Werner, Krieg, and Smola, 2007; Stunova and Vistejnova, 2018).

Multilayer engineered skin, similar to natural skin composition, is an even farer goal to reach with the actual technology (Morelli et al., 2023). Experimental use of cosmetics and drugs is another field where engineered skins are necessary, since restrictions are applied to animal use. To this purpose, 3D bioprinting represents a promising approach to obtain a source of biomimetic cellular skin substitutes for clinical and industrial applications. Natural biopolymers, like proteins (collagen, gelatin, albumin, thrombin, fibrinogen) and polysaccharides (chitosan, chitin, cellulose, alginate) are associated with biocompatibility, biodegradability and hydrophilicity. Their biological characteristics make them preferable for tissue engineering purposes (Agrawal et al., 2014; Bertassoni et al., 2014). Synthetic biopolymers include polylactic acid (PLA), polyglycolic acid (PGA), Poly (ε-caprolactone) (PCL), polylactic-co-glycolic acid (PLGA), poly-propylene fumarate, poly-anhydrides, polycarbonates, polyethers, polyurethanes, polyphosphazenes. 3D bioprinted skin holds great application potential to graft for wound healing, replacement of burned skin, and *in vitro* human models for experimental study of products and drugs (Vijayavenkataraman et al., 2016; Cubo et al., 2017).

## 6 Conclusion

Over the last decade there has been significant interest in the development of 3D tissue engineering constructs using bioprinting technologies. In particular, recent advances in extrusion-based bioprinting technologies and related materials have shown promise in the realisation of architecture, composition, structure and vascularisation of different types of tissues and organs. Even though initial work has focused on regenerative medicine applications, it is clear that extrusion-based bioprinting has also great potential in disease modelling applications. Bioprinted models can be the basis for understanding the underling biology mechanisms involved in disease progression and can help identify efficient therapeutic regimens.

Extrusion-based bioprinting is still at its infancy and requires substantial advances to reach its full potential. An outstanding challenge includes improving printing accuracy and speed. These currently critical shortcomings must be tackled through the development of innovative solutions whilst at the same time assuring that cell viability is not compromised by increases in shear stress, pressure and/or temperature. Extrusion-based bioprinting is increasingly being recognised as a viable technology for a wide array of biomedical and biotechnology applications. The advantages of extrusion-based bioprinting include high accessibility, low costs, and high printing accuracy. Extrusion-based bioprinting has also advantages over other biofabrication methods: it enables the production of more complex constructs, which better reflect the anatomic structures. It also offers an easier pathway towards automation, compared to other biofabrication technologies, thus paving the way to future mass-customisation of production. This latter aspect is facilitated by bioprinting being intrinsically suitable for producing constructs that are tailored to the individual needs of the patient.

Particularly interesting is the application of bioprinting to the fabrication of models capable of replicating diseased microenvironmental niches, e.g., cancer or myocardial infarction. Generating systems for complex and long-term dynamic cell culture would enable the study of individual or synergistic effects of biochemical and biophysical cues on disease initiation and progression. In this regard, developing disease-specific bioinks such as those derived from decellularised ECM of healthy and diseased tissues, could be a promising strategy to better mimic *in vivo* conditions. Similarly, the use of patient-specific cells could propel the understanding of the mechanism of diseases, which involve gene-associated changes on a personalised level.

Clearly, many challenges need to be overcome in extrusion-based bioprinting technologies and in the design of innovative bioinks, in order to enable the fabrication of higher-accuracy, higher-resolution and higher-stability constructs capable of supporting the most advanced applications. For example, the problem of replicating the proper vascularisation of bioprinted tissues is still partially unsolved, due to the very high resolution and accuracy requirements of the task. Similarly, the mechanisms of the immune response after the implantation of biofabricated constructs needs to be fully evaluated.
